# Protocol for the internet-based lifestyle intervention to eradicate obese frailty in prostate cancer survivors (iLIVE) randomized controlled trial: a type I hybrid effectiveness implementation trial

**DOI:** 10.1186/s12885-026-15851-4

**Published:** 2026-03-25

**Authors:** Wendy Demark-Wahnefried, Laura Q. Rogers, Lisa Zubkoff, Maria Pisu, Arthur Hung, Julie N. Graff, Joelle Hamilton, Nathan F. Dieckmann, Kerri Winters-Stone

**Affiliations:** 1 Clinical and Diagnostic Sciences, University of Alabama at Birmingham (UAB), Birmingham, USA; 2 O’Neal Comprehensive Cancer Center at UAB, Birmingham, AL USA; 3 Department of Medicine, UAB, Birmingham, AL USA; 4 Department of Radiation Medicine, Oregon Health & Science University, Portland, OR USA; 5 School of Medicine, Oregon Health & Science University, Portland, OR USA; 6 School of Nursing, Oregon Health & Science University, Portland, OR USA; 7 Knight Cancer Institute, Oregon Health & Science University, Portland, OR USA; 8 Division of Oncological Sciences, Oregon Health & Science University, Portland, OR USA

**Keywords:** Obesity, Frailty, Prostatic neoplasms, Intervention, Internet, Randomized controlled trial, Sarcopenia, Older, Diet, Physical activity

## Abstract

**Background:**

Obese frailty is a growing national problem as trends towards aging and obesity converge. This problem is especially acute in men with prostate cancer treated with androgen deprivation therapy (ADT). ADT is the backbone of therapy for advanced prostate cancer, a disease which affects mostly older men. But, ADT accelerates muscle loss and gains in adiposity which threaten physical functioning and quality of life. Diet and resistance training may mitigate obese frailty but have rarely been combined in an accessible delivery format.

**Methods:**

The Internet-Based Lifestyle Intervention to Eradicate Obese Frailty in Prostate Cancer Survivors (iLIVE) Randomized Controlled Trial (RCT) aims to mitigate obese frailty among men with prostate cancer treated with ADT. Participants (N=250) are being recruited across North America to participate in this RCT that is implemented entirely over the internet. Eligibility criteria include body mass index >25 kg/m2 and > 2 frailty symptoms [illness, fatigue, inactivity, weakness, slowness]). Consented participants undergo assessments that include: online surveys (medical status, falls, physical activity, quality-of-life, healthcare utilization); dietary recalls (kilocalories, diet quality); body weight (Aria® scales); physical activity (Fitbits®); 4) internet-based physical performance (chair stand, timed up-and-go, gait speed tests); and 5) muscle mass (D3 creatine dilution). Participants are then randomized to one of two 6-month interventions: 1) Enhanced Usual Care (EUC): Encouraged to continue Aria® scale and Fitbit® use with monthly emails of publicly available diet- and exercise-related information; or 2) iLIVE: Same as EUC, plus website access to weekly serialized sessions on weight loss and diet quality and thrice-weekly live remote supervised group resistance training sessions. Assessments are repeated at 6-months; at 12-months, questionnaires are repeated and data from digital devices are captured. Intent-to-treat analysis will compare EUC vs. iLIVE (baseline-to-6-months) using a composite measure of obese frailty as the primary outcome. Designed as a type I hybrid-effectiveness implementation trial, data on intervention feasibility (including safety), acceptability, adoption, appropriateness, fidelity, sustainability, and cost are assessed, tracked, and analyzed.

**Discussion:**

The iLIVE RCT is implemented exclusively over the internet and provides a blueprint for other highly scalable lifestyle interventions with potential for rapid translation into nationwide practice.

**Trial registration:**

ClinicalTrials.gov: NCT06011499 (5.31.2025: https://clinicaltrials.gov/study/NCT06011499).

**Supplementary Information:**

The online version contains supplementary material available at 10.1186/s12885-026-15851-4.

## Background

Approximately 60% of American men age 65 and older have prostate cancer, making it the most common malignancy aside from non-melanoma skin cancer in the nation [1]. Nearly half of these men are prescribed androgen deprivation therapy (ADT) [2]. While ADT is necessary to assure cancer control among men with more progressive disease [3], it comes at a price, specifically, the exacerbation of obese frailty (a condition characterized by high fat mass concurrent with low muscle mass) already prevalent among older adults, and a condition that increases the risk of comorbidity and falls, and that reduces physical functioning and overall quality of life [4–8]. Unfortunately, the prevalence of obese frailty remains high even after ADT is discontinued, further amplifying its impact [8].

While “obese frailty” is an emerging concept, the co-occurrence of the obesity epidemic with the aging of the American population and attendant increases in disability have been noted for decades. In 1994 and in a sample of > 3,000 elders, Galanos et al. [9] noted that the risk of functional impairment was significantly higher among those with body mass indexes (BMI) that were < 21.1 kg/m^2^ (Odds Ratio [OR]: 1.34, 95% Confidence Interval [95% CI: 1.04–1.74]), and even greater among those with BMI’s > 31.0 kg/m^2^ (1.43 [95% CI: 1.10–1.86]. As the American public has become older and heavier, reporting on the problem of sarcopenic obesity (a related condition) has been the focus of a handful of interventional studies [10, 11]. A review by Starr and colleagues suggests that multi-behavior interventions that include both dietary modification to reduce caloric intake and increased exercise (especially multifaceted programs involving aerobic, flexibility, and resistance training) offer the best strategy for managing sarcopenic obesity in the broader population of older adults [12]. Despite the overwhelming need to address obese frailty (which not only encompasses the body composition changes inherent with sarcopenic obesity, but also the resulting insult to physical functioning) in special high-risk clinical populations, such as prostate cancer patients on ADT, as well as the population at large, there is a dearth of scalable interventions - interventions that are safe, efficacy-proven, expertly led, and which can be scaled broadly with fidelity to meet the growing needs in this nation.

This paper describes the intervention and the methods of the Internet-Based Lifestyle Intervention to Eradicate Obese Frailty in Prostate Cancer Survivors (iLIVE) Randomized Controlled Trial (RCT). This National Cancer Institute-supported trial is a collaborative effort between investigators at the Oregon Health and Science University (OHSU) and the University of Alabama at Birmingham (UAB). iLIVE is currently enrolling hundreds of men across the country to test the impact of a 24-week web-based intervention that was adapted from a live remote supervised group resistance training program [13], and tested in the GET FIT Prostate trial [14], and then combined with a serialized, web-based diet (weight loss) intervention tested in the AiM, Plan, and act on LIFestYles (AMPLIFY) trial [15]. The primary aim of the iLIVE RCT is to compare the combined resistance training and weight loss intervention to enhanced usual care (i.e., an arm receiving identical self-monitoring devices and access to publicly-available physical activity and diet information) on obese frailty, with secondary aims to explore between-arm differences regarding individual components of obese frailty (e.g., body weight, muscle mass, fatigue, slowness, weakness, and inactivity) and intervention implementation outcomes (e.g., feasibility [including safety], acceptability, cost, sustainability), and to investigate potential effect modifiers to identify those men who benefit the most from the iLIVE intervention. A unique feature of iLIVE is that all components are delivered and assessed fully through remote means.

## Methods/design

### Design and settings

iLIVE is a 2-arm, single-blinded RCT that relies on a single Institutional Review Board Protocol administered by the OHSU Knight Cancer Institute (25281) with approval by UAB (300011042). Designed with the anticipation that, if found acceptable and effective, iLIVE could progress to future larger-scale implementation effectiveness trials, the RCT was designed under a Type I Hybrid Effectiveness-Implementation framework that simultaneously assesses intervention effectiveness and implementation factors [16]. iLIVE is registered on ClinicalTrials.gov (NCT06011499) and is being conducted throughout North America. Active solicitation via physician referral, patient portal prompts and letters of invitation with telephone follow-up are underway at OHSU (and through their liaison with the Oregon State Cancer Registry) and UAB, with nationwide outreach conducted through national and regional prostate cancer support groups, as well as a study website (https://ilive4health.org/welcome). Since all study activities occur online, men can be recruited from anywhere across the continent through various strategies, but all consenting is conducted electronically by the OHSU study team. Signed informed consent (in which the risks of dietary modification and physical activity are detailed, and participants acknowledge these risks and assume potential related-costs) is obtained from all trial enrollees prior to data collection via Adobesign^®^ (San Jose, CA); study ID numbers that assure that the collection of data is deidentified are assigned at that time.

### Eligibility

Men expressing interest in trial participation are screened via telephone by iLIVE study staff to assure that the following eligibility criteria are met: (1) age 18 and above; (2) diagnosed with prostate cancer with completion of radiotherapy, chemotherapy and/or surgery; (3) received > = 6 months of ADT any time in the past 10 years; (4) BMI: 25-<50 kg/m^2^ (with no evidence of unintentional weight loss of > 5% within the last year); (5) not currently engaging in a structured diet or resistance training exercise program; (6) absence of health conditions that potentially affect weight, ability to pursue moderate physical activity, and/or the ability to participate in the intervention or its assessments (e.g., Cushing’s syndrome, uncontrolled hyper/hypo-thyroidism, debilitating stroke, another active invasive malignancy; not fluent in English); and (7) willingness to be randomized and participate fully in the RCT. Men who have yet to finish their primary treatment are placed on a waitlist and activated upon completion, and because muscle mass is being assessed via the D3 creatine dilution method [17], men taking creatine supplements are instructed to pause them for one month before undertaking baseline and 6-month assessments in order to be considered for the trial. Healthcare providers for all potential enrollees are contacted to confirm diagnosis and ADT history. Individuals who meet al.l criteria are then scheduled for a videoconference assessment to assure that their home internet is sufficient for intervention delivery and assessment; and to gather further evidence of frailty by meeting three or more criteria of the Fatigue, Resistance, Ambulation, Illnesses, & Loss of Weight (FRAIL) scale unrelated to weight, i.e., self-reported fatigue, inactivity, or illness and/or prolonged completion (compared to age-related norms) of objective performance testing of the 5-repetition chair stand and the 4-meter walk that suggest weakness or slowness [18], respectively.

### Measures

Individuals deemed eligible are then scheduled for a video conferenced baseline appointment and express mailed the following supplies: (1) a Wi-Fi-enabled Aria^®^ scale and Fitbit Inspire 2 (Google, LLC, Mountain View, CA)(to assess body weight and physical activity); (2) a 30 mg capsule of D3 creatine and urine test strip (to assess muscle mass); and (3) printed instructions. Other equipment needed for online testing (e.g., tablet, webcam, Bluetooth speakers) is included as needed on a case-by-case basis. Participants are emailed links to assist with Fitbit^®^ and Aria^®^ scale set-up (https://360.articulate.com/review/content/994b1496-5e1b-44ac-86ab-0bc1831c5c02/review) and YouTube video instructions for D3 creatine assessments (https://www.youtube.com/watch?v=xP9JnG3qosY&t=1s). Participants also are encouraged to call the study office upon receipt of the package so that a staff member can review the materials, answer questions and facilitate the connection between the scale and Fitbit with the Fitabase database that is managed by study staff. All study databases used by this trial are only accessible by staff members who have completed human subjects training and are password protected and managed behind secure firewalls. All data (except Fitabase and D3 creatine-related data) is logged directly into REDCap^®^ (Nashville, TN) for which the programming assures minimization of missing data and out-of-field observations. All study assessors are trained and blinded with regard to the study condition.

**Fig. 1:**
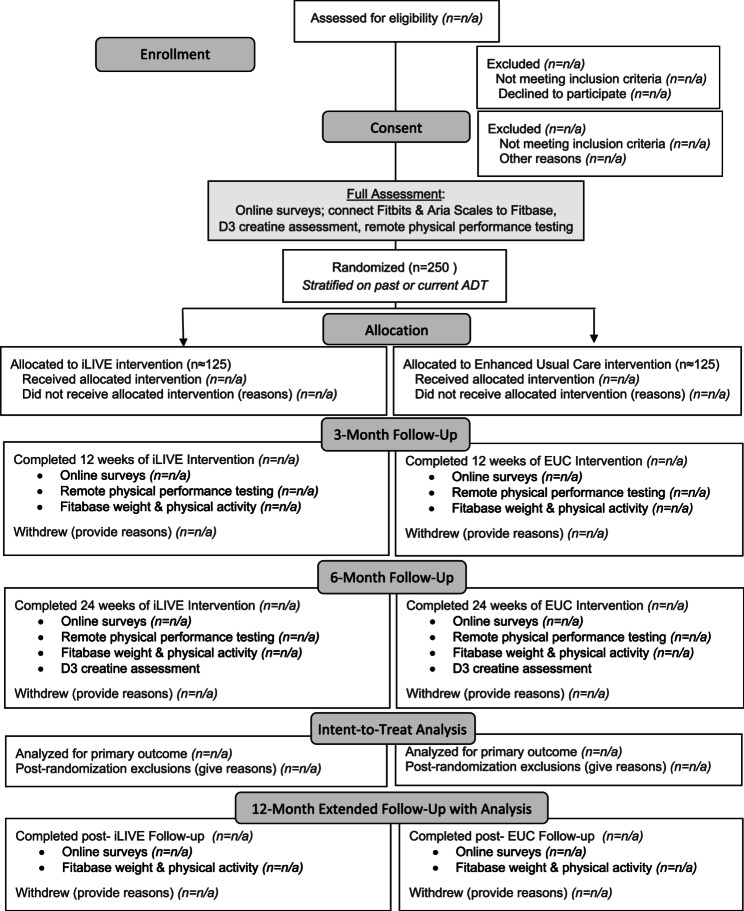
iLIVE study design and flow diagram Legend: The intention-to-treat analysis will use all data from randomized participants who are not excluded due to death or the development of other serious subsequent conditions that affect body weight and function

At baseline, participants complete an online survey to report or verify demographic and health data including age, race, ethnicity, income, years of education, marital status, and height (which is assumed to stay constant over the study period). The Charlson Comorbidity instrument also is embedded within this survey [19]. Additional data are collected at baseline and 3-, 6- and 12-month follow-up (with the 6-month follow-up serving as the primary timepoint). See Fig. 1 that depicts study flow.

### Primary outcome

A reduction in the Obese Frailty Phenotype (OFP) score serves as the primary outcome and it is defined as clinically significant weight loss of 5% AND clinically meaningful change in 2 or more frailty components, i.e., weakness, slowness, fatigue, inactivity, and/or illness (see below). The OFP is a dichotomous binary score that indicates improvement when both elements are met (yes) or not (no).

#### Body weight

The Aria^®^ scale directly relays data on each participant’s body weight throughout the study period. Email reminders encourage each participant to weigh themselves (in the morning, without shoes and in light clothing) throughout the study and weights are explicitly taken during the time of video conference assessments. A weight loss of *≥* 5% from baseline to 6-months is considered clinically significant weight loss (see criterion #1 above) [20], and contributes to the OFP composite score.

#### Physical inactivity

As with the Aria^®^ Scale, the Fitbit^®^ also relays data continuously throughout the study period; kcal/week spent in moderate-to-vigorous physical activity (MVPA) is captured at baseline, 3-, 6-and 12-month follow-up timepoints. In addition, the widely-used, Community Health Activities Model Program for Seniors (CHAMPS) physical activity questionnaire is administered online at identical timepoints [21]. For both objective and subjective measures a cut point of *≥* 383 kcals of MVPA/ week will signal an absence of frailty (and will be used in calculating the OFP score); however, in instances in which a discrepancy occurs, results from the self-report will used [21].

#### Sarcopenia

The D3 creatine dilution method, as implemented and analyzed in the Osteoporotic Fractures in Men (MrOS) study [17, 22], will provide data on muscle mass. Participants will ingest a 30 mg D3 creatine capsule and then urinate on a test strip on the second void upon waking and after at least a 12-hour fast, 3–6 days later. Test strips from baseline and 6-month follow-up will be stored in the participants’ freezer until collection at 6-months (either through staff pick-up or via express mail using chilled biospecimen protocols). Given the complex logistics which require additional mailing, staff follow-up (i.e., reminder calls to ingest the capsule and then use the test strip in a fasting state), participant burden, and expense, this measure is only conducted at baseline and 6-months. A cutoff of *≥* 10.75 kg/m^2^ is suggestive of the absence of moderate sarcopenia [23], and hence the absence of frailty for the OFP score (above).

#### Weakness

A timed 5-repetition chair stand time of < 12 s is reported as significantly reducing the risk of falls by roughly 58% [24], thus suggesting the absence of frailty and a factor taken into account in the OFP score (above). This measure will be performed remotely at baseline, 3- and 6-months using a protocol with established excellent intra-rater reliability, i.e., (Intra Class Coefficients [ICC] = 0.92, 95% Confidence Interval [CI] 0.84–0.96) [25].

#### Slowness

As with weakness, this component of frailty (i.e., 4-m walk) will be measured remotely at baseline, 3- and 6 months using a protocol in which the established intra-rater reliability is good, i.e., ICC: 0.87, 95% CI 0.71–0.94 [25]. Usual gait speed will be assessed twice at each timepoint with the fastest observation recorded for subsequent analysis. Values *≥* 1.0 m sec^− 1^ will be viewed as an absence of frailty for the OFP score (above), as comparable gait speeds have been reported as protective in association with falls and a loss of independence [26].

#### Fatigue

This construct of frailty will be measured online via self-report at baseline, 3-, 6- and 12-months using the 4-item Short Form 36 (SF36) vitality subscale [27]. Scores of *≥* 50.00 (normed) will be used for participants aged 50–64 years or scores *≥* 40.00 (normed) will be used for those aged 65 + years as indicating a non-frail state (and used for the OFP score) [28].

### Secondary outcomes

#### Additional measures of physical performance/function

Select components of the Senior Fitness Battery, i.e., the Timed Up and Go (TUG), a widely-used measure of functional mobility and agility, as well as side-by-side, semi-tandem, and tandem standing balance testing also are performed at baseline, 3- and 6-month follow-up via video, given strong concordance with in-person testing [25, 29]. The balance tests can also be combined with the walk speed and chair stand tests to construct the short physical performance battery which has strong predictive validity for increased risk of disability, nursing home admission, and mortality as scores drop below 10 [30]. In addition, the 10-item, SF36 Physical Function subscale is embedded into the online survey at baseline, 3-, 6-, and 12-months [27].

#### Dietary intake

Both calorie intake and diet quality serve as secondary outcomes; hence at baseline and 6-month follow-up, unannounced 2-day diet recalls of 1-weekday and 1 weekend day are collected by a registered dietitian via telephone and entered into the NCI-developed ASA24 [31]. The Healthy Eating Index – 2020 will be used to calculate diet quality [32].

#### Global quality of life

In addition to collecting data on specific subscales of the SF-36, participants also provide online responses to the EuroQol-5 Dimension (EQ-5D) at baseline, 3-, 6- and 12-months: the 5 dimensions are mobility, self-care, usual activities, pain/discomfort, and anxiety/depression. The EQ-5D was chosen as it provides a low burden measure of QOL [33, 34], and can be used to calculate Quality of Life Adjusted Years (QALYs) for the cost-effectiveness analysis.

#### Health care utilization

At baseline, 3-,6-,9- and 12-month follow-up all participants will be asked to report physician and emergency room (ER) visits, and hospitalizations using the instrument of Ritter et al. [35] which demonstrates test-retest reliability 0.76–0.97, low discrepancy with provider records, and no variation by demographics or health status of respondents. These data (in addition to QALYs – see above) will be used for the cost analysis.

#### Falls and related injuries

Due to the sarcopenic nature of the study population, falls and their related injuries are measured frequently, i.e., at baseline (retrospectively, with respect to the previous year) and monthly throughout the study period, using monthly fall reports used in previous fall prevention interventions [14, 36].

#### Safety

Participants are encouraged to report adverse events as they occur but are also queried monthly throughout the 12-month study period. The description of the event is collected, along with details on the medical care received, and whether the participant attributed it to exercise associated with either study intervention or whether they attributed it to dietary changes they were undertaking to reduce their caloric consumption, e.g., indigestion due to higher intakes of plant-based foods. These events are reviewed quarterly by the study team who are blinded to arm assignment and who classify events as serious (i.e., life threatening, permanently disabling, and/or resulting in overnight hospitalization) or non-serious, and attributable, possibly attributable, or non-attributable to the intervention. Obvious serious events are evaluated within 72-hours by the trial MPI’s with assistance from co-investigators with certified medical training and reported to the OHSU Institutional Review Board (IRB). All events are reviewed annually by the OHSU IRB, as well as an independent Data Safety and Monitoring Board (comprised of a biostatistician, clinician-scientist, and a patient advocate) to confirm adherence to safety standards and assess any imbalances between study arms. No interim analyses are planned.

### Implementation outcomes

Because the iLIVE intervention has high potential for scalability and, if proven efficacious, could help substantial numbers of men, the iLIVE trial was designed using a Hybrid Type I Implementation framework [16]. Select evaluation criteria proposed by Proctor et al. [37] informed data collection in the following intervention implementation areas: feasibility; cost; acceptability; adoption; appropriateness; fidelity; and sustainability.

As described under Secondary Outcomes, some measures serve as independent outcomes but also contribute to broader implementation domains. For example, safety is an important independent outcome, but, when evaluated along with process data gathered on intervention engagement (i.e., % of website sessions completed [obtained from website analytics], use of scales and Fitbits^®^ [obtained from Fitabase^®^], and class attendance logs), as well as retention from study tracking, the end result is a more comprehensive evaluation of feasibility. Likewise, health care utilization and QALYs and costs associated with intervention delivery will be used in a thorough cost-analysis to assess cost-effectiveness of the intervention. To evaluate other implementation domains, a combination of quantitative data (from recruitment or tracking databases, website or device analytics, or participant surveys), along with qualitative data (from semi-structured interviews with participant subsamples or stakeholders [health care providers and health system administrators]) provides a comprehensive approach to evaluate other domains. Examples are as follows.

#### Acceptability

Recruitment databases yield enrollment rates (total and in specific age, race/ethnicity, or residential subgroups) and percent refusals or ineligibles by reason, whereas semi-structured interviews among refusers and enrollees provide qualitative data on ways to improve future enrollment and retention. Acceptability also is assessed among providers and administrators.

#### Adoption

Recruitment databases yield sources of referral, tracking databases, or data from website or device analytics provide quantitative data on withdrawal and adherence, whereas semi-structured interviews among dropouts, health care providers, and health system administrators provide qualitative data on barriers/facilitators to referral or participation.

#### Appropriateness

Post-intervention surveys (at 6 months) query all participants on the relevance and fit of the interventions using quantitative means, whereas semi-structured interventions completed on a subset of participants provide more detail on suggestions for future programming. Interviews among providers and health system administrators delve into how iLIVE aligns with the extant mission and fits into existing services. 

#### Fidelity

Data from website or device analytics, as well as training logs and fidelity checklists provide quantitative data on intervention delivery, whereas semi-structured interviews among participants provide qualitative data on program quality and suggestions for improvement. 

#### Sustainability

All participants are encouraged to continue with interventions beyond 6-months and their engagement with websites, devices, and in the case of the experimental group continued exercise session attendance, is monitored from 6 to 12 months to gauge sustainability; semi-structured interviews with health care providers and health system administrators provide qualitative data to inform future integration with clinical care.

Semi-structured interviews are conducted over the telephone by trained moderators using interview guides tailored toward the patient subsample or type of stakeholder and using in-depth interviewing methods [38].

### Randomization and interventions

Upon completion of baseline data collection, participants are block randomized (size = 6) by timing of ADT (current vs. past). Randomization sequences are generated by the study biostatistician who has no contact with participants, and who then informs the study coordinator about assignment to the iLIVE intervention (iLIVE) or enhanced usual care (EUC) arms. Both arms receive an emailed “Welcome” message informing them of their status along with pertinent links to websites and equipment. Because self-monitoring is a key construct of behavior change [39, 40], both arms are encouraged to continue Fitbit^®^ and Aria^®^ scale use and use the open access resources available through MyFitnessPal, Inc. (https://www.myfitnesspal.com/). iLIVE is designed as an adjunct to standard care and not interfere with its delivery. The interventions and assessments are conducted exclusively via the internet.

#### iLIVE intervention

The iLIVE intervention is an amalgam of previous interventions that have shown efficacy in improving diet quality and promoting weight loss, as well as increasing physical activity, especially aimed at improving strength and physical function, i.e., resistance training, among cancer survivors. The message library developed for the efficacy-proven Reach-out to Enhance Wellness (RENEW) intervention which was originally delivered via tailored print materials and telephone counseling to 641 breast, prostate and colorectal cancer survivors [41–43], was first adapted for web-use in the Daughters, dUdes, mothers and othErs Together (DUET) trial among 112 cancer survivors and chosen partners [44, 45], as well as the AiM, Plan and act on LIFestYles (AMPLIFY) trial (*n* = 351) [15, 46, 47], both of which confirmed efficacy. The web-based interventions which provide content that is refreshed routinely via tips of the day, weekly interactive sessions (tutorials), features to track dietary behaviors and body weight, and over 100 tools (e.g., meal plans, grocery lists, cooking videos) were adapted exclusively to meet the needs of prostate cancer survivors on ADT. Likewise, previous functional resistance training programs found effective in improving strength and reducing falls in supervised, facility-based group classes [48–50], were adapted to live remote instruction using standard videoconferencing software (e.g., Zoom), and led by a trained instructor who leads the exercises, modifies exercises for safety and tolerance, provides feedback on proper form, and offers encouragement. A second staff member is either online or on call during exercise sessions to assist with videoconferencing issues and/or safety monitoring. The resistance training program starts with several weeks of mobility and stability exercises designed to improve efficiency and safety of functional exercises that are the core of the functional strength training program. These mobility exercises are then integrated into the warm-up moving forward. The functional strength training program consists of a 60-minute session with a 5–10-minute warm-up, 45 min of functional resistance training, followed by a 5–10-minute cool down. The routine set of functional exercises consists of wall-sits, chair stands, step-ups, push-ups, rows, and core (e.g., plank, bird dog, bridge, dead bug) that are done during each session. As with prior facility-based programs, weighted vests are used to provide overload - an innovative method to progressively increase resistance in the lower body to improve musculoskeletal function without safety risks related to balance disruption that can occur with handheld weights. Given that the vest has multiple pockets distributed evenly around the torso with each holding a ½-lb weight, exercise intensity can be adjusted slowly and in an ergonomically efficient and safe way. The volume of resistance training progresses from the low-to-high moderate intensity range over the 24-week intervention using progression outlines in which vest weight (intensity) is gradually increased from 4% to 15% of body weight, while sets increase from 1 to 3 sets of 10–15 reps (as weight increases the rep range will decrease) [36, 48, 51]. Participants login thrice weekly, for 75-minute sessions that open 15 min prior to and after the exercise session to provide men with time and opportunity for personal interactions - a key element that provides valued peer support that can optimize adherence and retention. Social support, along with self-efficacy and reducing barriers to behavior change are key constructs of Social Cognitive Theory [39, 40], which serves as a unifying behavioral framework for both the physical activity and diet-related components of the iLIVE intervention.

#### Enhanced Usual Care (EUC)

As with men assigned to the iLIVE intervention, participants assigned to this arm receive the self-monitoring devices described above, as well as links to consensus-driven diet and exercise information in the public domain, such as American Cancer Society’s Guidelines for Cancer Survivors (https://www.cancer.org/health-care-professionals/american-cancer-society-survivorship-guidelines.html), the American College of Sports Medicine’s Moving Through Cancer (https://www.exerciseismedicine.org/eim-in-action/moving-through-cancer/) and the American Institute for Cancer Research New American Plate (https://www.aicr.org/cancer-prevention/healthy-eating/new-american-plate/). Monthly newsletters posting links to this information (identical links which are embedded within the website content that comprises the experimental iLIVE intervention) are provided to encourage trial retention, and to ensure that all trial participants receive the standard of care for lifestyle factor guidance in accordance with recommendations of the American Society of Clinical Oncology and the National Comprehensive Cancer Network [52–54].

### Statistical analysis

#### Power and sample size calculation

The sample size requirements for this RCT were based on estimates obtained from an ongoing trial [36]. We estimated that 40% of men assigned to the iLIVE intervention vs. 10% of those assigned to usual care will demonstrate clinically significant improvements from baseline to 6-month follow-up in the obese frailty phenotype (defined as clinically significant weight loss of 5% and clinically meaningful change in 2 or more frailty components, i.e., weakness, slowness, fatigue, inactivity, and/or illness). However, we powered this trial to detect smaller effects that are likely still clinically meaningful. Assuming alpha=0.05 (two-tailed) and power=0.80, a sample size of 200 complete cases (100 in each group) would be required to detect proportional differences in the 18–20% range. Assuming an attrition rate of ~ 20%, the total sample size required is *N* = 250 (*n* = 125 in each group). If attrition is lower than expected, we will need to randomize fewer participants to achieve the desired sample size. Additionally, this sample size results in even more statistical precision when examining secondary outcomes that yield continuous data.

Sample sizes for the implementation analysis are based on estimates of achieving thematic saturation [55, 56]. Here, we anticipate interviewing 30 prostate cancer survivors (6 who refuse trial participation, 12 who enroll but drop out before the end of 6 months, and 12 who complete their 6-month assessment). Maximum variation sampling will be done based on study group allocation (enrollees only), age (< 75 vs. 75+) and region of the country (west vs. east). Plans also include interviewing eight oncology clinical care team members (different types of oncology providers and nurses) and eight health system administrators (Chief Executive Officers, etc.).

#### Planned data analysis

##### Quantitative data analysis

Once data collection is complete, study retention will be examined to determine between-arm differences and explore potential differences between drop-outs and study completers. Study data will be examined using standard descriptive statistics and graphics to check for distributional assumptions, the presence of outliers, and patterns of missingness to inform final modeling strategies (e.g., the use of model-based maximum likelihood estimation) [57]. All analyses will be conducted using an intention-to-treat approach (using all data from randomized participants who are not excluded due to death or the development of other serious subsequent conditions that affect body weight and function) using R v3.3.2171 9 (R foundation, Vienna, Austria) and Mplus v7.31172 (Los Angeles, CA) software. In addition to traditional significance testing, Cohen’s d or odds ratio effect sizes (with 95% confidence intervals), as appropriate, will be estimated for change in primary and secondary endpoints across time. Model diagnostics (e.g., examining residual/predicted value plots) will be used to confirm statistical assumptions have been met. In the case of violations, more robust estimation procedures, such as nonparametric bootstrapping, will be employed as appropriate.

Standard logistic regression models will be used to test between-arm differences for both the primary and secondary binary outcomes. Complementary analyses will be conducted on each continuous frailty component separately using a linear mixed effects modeling approach implemented in the R lme4 package. Contemporary methods will be used to control for false discovery rates when testing secondary outcomes [58]. Durability of the effect of iLIVE vs. the EUC intervention after the structured program stops (i.e., at 6 months), will be explored in a limited number of outcomes (e.g., body weight, physical activity and self-reported measures of fatigue and quality of life using baseline to 12-month data). Time will be modeled either as a fixed effect with two contrasts comparing baseline to 6-month, and baseline to 12 month follow up or as a piecewise model with separate estimates of change between baseline and 6 months and 6 months and 12 months. The primary effects of interest will be the cross-level interactions between intervention group (fixed binary predictor) and the time contrasts. Significant interactions will indicate that outcome change across time differs between the intervention groups. Potential moderators (e.g., age, BMI, time from diagnosis) will be explored by adding these terms to the models and testing for interaction. These analyses will allow us to determine the characteristics of patients who have the highest likelihood of change. We also have experience using Latent Class Analysis on longitudinal change estimates to discover clusters of intervention responders [59].

##### Cost analysis

In addition to the descriptive calculation of costs, a within trial cost-effectiveness analysis will compare the implementation costs net of health care cost savings (if any) to the effectiveness of iLIVE vs. EUC on the gain in QALYs calculated using utility weights derived from the EQ-5D [34, 60]. Health care savings will be determined by comparing health care costs derived from self-reported health care utilization data between the two study arms [35]. Regression analysis, or non-parametric bootstrap techniques if data are skewed, will estimate differences in QALYs and health care costs between iLIVE and EUC [61, 62]. An incremental cost effectiveness ratio will be calculated and compared to common thresholds to determine if iLIVE is cost-effective compared to EUC [63, 64].

##### Qualitative data

A general inductive analysis approach will be undertaken [65]. Transcription of interviews will occur using standardized rules and reviewed for accuracy; data will be managed using NVIVO software (Lumivero, Denver, CO). Coding schemes will be developed to uncover emergent themes with iterative review by multiple team members to ensure findings are robust. Through this process, rich datasets are created that can guide future implementation and possibly refine current methods used for referral.

## Discussion

The iLIVE trial is aimed at mitigating obese frailty, a particularly acute issue in the rising numbers of American men diagnosed with prostate cancer who are treated with ADT but is also a major public health issue in the burgeoning numbers of older adults in the nation. The interventions that are tested, as well as the means to evaluate them are highly scalable and the trial was designed from the start with the ultimate goal of broad dissemination. Hence the methods to evaluate this trial are not only geared toward scientific rigor, but the trial itself is firmly rooted in implementation science and will benefit from the scaffolding and benchmarks that will accelerate potential translation to both clinical practice and public health.

The iLIVE trial features several unique elements. First, the interventions being evaluated are delivered exclusively via remote means, including remote assessments and live exercise training using videoconferencing software. Of the RCTs that are listed under PubMed, less than 2% fall within this category, though the percentage of distance-medicine based interventions has increased in the post-COVID era. Roughly half of the published interventions are delivered via eHealth-based approaches. However, even though more interventions are being delivered via the internet, most RCTs still require in-person assessments within the clinic setting. The iLIVE trial is a rare example where both the intervention and the assessments are done exclusively via the internet; thus, overcoming barriers of time and travel that are frequently cited for low trial participation [66, 67], especially among older populations [68]. Thus, while previous research suggests that older adults may lack computer literacy and access to the internet, these issues can be overcome [69]. Data from Pew surveys, shows that individuals age 65 and older have the fastest growing rates of computer use and home broadband access, with 2024 rates now being 90% and 70%, respectively [70]. Therefore, iLIVE represents the new frontier of care delivery and clinical trial design – one that tests scalable interventions that can be delivered and assessed with fidelity and lower cost than traditional means, and that can be scaled broadly to larger, more diverse populations. These design features increase the probability of iLIVE being accelerated to larger effectiveness-implementation trials, and eventual translation into clinical practice. Hence, another feature of the iLIVE protocol is that it is designed under a Type I Hybrid Effectiveness-Implementation framework and thus provides a blueprint for other trials that also seek more rapid translation to practice.

## Supplementary Information

Supplementary Material 1.

## Data Availability

Upon completion of the iLIVE RCT, the published primary outcomes paper will be made available to all study participants (prostate cancer survivors, healthcare providers and administrative stakeholders). Results also will be shared at professional scientific meetings and support groups. The deidentified datasets generated during and/or analyzed during the present study will be available from the corresponding author upon reasonable request.

## Abbreviations

ADT: Androgen Deprivation Therapy
AMPLIFY: AiM, Plan, and act on LIFestYles
BMI: Body Mass Index
CHAMPS: Community Health Activities Model Program for Seniors
CI: Confidence Interval
DUET: Daughters, dUdes, mothers and othErs Together trial
EQ-5D: EuroQol-5 Dimension
ER: Emergency Room
EUC: Enhanced Usual Care
FRAIL: Fatigue, Resistance, Ambulation, Illnesses, & Loss of Weight
IRB: Institutional Review Board
ICC: Intra Class Coefficient
iLIVE: Internet-Based Lifestyle Intervention to Eradicate Obese Frailty in Prostate Cancer Survivors
MVPA: Moderate-to-Vigorous Physical Activity
MrOS: Osteoporotic Fractures in Men
OFP: Obese Frailty Phenotype
OR: Odds Ratio
OHSU: Oregon Health and Science University
QALY: Quality of Life Adjusted Year
RCT: Randomized Controlled Trial
RENEW: Reach-out to Enhance Wellness trial
SF36: Short Form 36
TUG: Timed Up and Go
UAB: University of Alabama at Birmingham
